# Mnemons: encoding memory by protein super-assembly

**DOI:** 10.15698/mic2014.01.134

**Published:** 2014-02-25

**Authors:** Fabrice Caudron, Yves Barral

**Affiliations:** 1 Institute of Biochemistry, Department of Biology, ETH Zurich, Otto-Stern-Weg 3, 8093 Zurich, Switzerland.

**Keywords:** mnemons, memory, pheromone response, Whi3, super-assemblies, budding yeast

## Abstract

Memory is mainly understood as the recollection of past events. The human brain and its simplest unit, the synapse, belong to the places in which such memories are physically stored. From an experimental point of view, memory can be tested in humans by recall. However, in other organisms, memory is reflected in its use by individuals to learn about and adapt their behavior to their environment. Under this criterion, even unicellular organisms are able to learn from their environments and show the ability to adapt their responses to repeating stimuli. This indicates that they are able to keep track of their histories and use these traces to elaborate adapted responses, making these traces akin to memory encodings. Understanding these phenomena may even help us to dissect part of the rather complex molecular orchestration happening in our synapses. When exposed unsuccessfully to mating pheromone, i.e. when mating does not happen, budding yeast cells become refractory to the mating signal. This refractory state is restricted to the mother cell and not inherited by the daughter cells, even though it is stable for most if not the entire life span of the mother cell. Interestingly, both stability and asymmetric segregation of the acquired state are explained by the molecular mechanism underlying its establishment, which shows important analogies and distinctions to prions. Here we discuss these similarities and differences.

## A Mating Refractory State

The budding yeast mating pathway has been extensively studied such that we have reached a detailed view of the molecular events happening, from receptor binding by pheromone to arrest in the G1 phase of the cell cycle. However, how cells respond to sustained exposure to pheromone without being able to successfully mate was not known. Upon constant exposure to pheromone in a microfluidic chamber, we observed that after their initial response and the formation of a shmoo, yeast cells escaped the G1 arrest and formed buds. At the next G1 they did not resume shmooing and kept budding. Therefore, yeast cells become refractory to pheromone, and once this state is established, it is as stable as a memory. Indeed, this refractory state is stable over potentially the entire life-span and does not need pheromone for its maintenance. Furthermore, yeast cells become refractory even before escaping the initial arrest, supporting the notion that they memorize the occurrence of pheromone exposure rather than perpetuating the process of escaping pheromone *per se*.

## Whi3 based super-assemblies

What is the molecular basis of this memory? Taking advantage of the knowledge of the mechanisms driving the G1 to S transition, we first identified the G1 cyclin Cln3 as a key player for imposing the refractory state. An inhibitor of Cln3 activity, the mRNA binding protein Whi3, represses the establishment of the refractory state by capturing and repressing translation of the Cln3 mRNA. Our data suggest that at some point after pheromone exposure, Whi3 is inactivated, releasing Cln3 activity. The presence of two domains rich in glutamine and asparagine (Q/N domains) has been recently highlighted in Whi3 by the group of Susan Lindquist. These domains are predicted to be able to adopt different conformations, like prions, which could in turn change the function of Whi3. We found that indeed Whi3 changes conformation during pheromone response, leading to its congregation into super-assemblies. In these assemblies, Whi3 loses its repressive function on Cln3 synthesis, allowing entry into a new cell cycle and the establishment of the pheromone refractory state.

## A case similar to prions?

The regulation of Whi3 through its Q/N rich domains is very reminiscent of prions. In fact, these domains were identified because they resemble the domains required for prion conversion of the yeast canonical prions Sup35, Ure2, Rnq1 and New1. Similarly to prions, the Q/N domains of Whi3 drive the assembly of proteinase K resistant species, suggesting that Whi3 adopts a distinct structural conformation. Prion conversion is regulated by chaperones and the protein disaggregase Hsp104. Whi3 super-assembly and the memory it helps to encode are also controlled by both the Hsp70 family member Ssa1 and to a lesser extent Hsp104, suggesting that the machineries controlling proteostasis are similarly important for controlling the conversion of Whi3 as for that of prions. In many cases, we observed a recruitment of Ssa1 to Whi3 super-assemblies. More importantly, a feature of prions is that once formed they are stable beyond the lifespan of individual proteins. Whi3 assemblies share this, as once cells become refractory to pheromone, they remain so for their entire life span.

## Mnemons differ from prions

Is Whi3 a new prion? No, it lacks one critical feature to be called a prion: Whi3 super-assemblies are asymmetrically inherited by the mother cell only and the trait encoded by the converted protein does not spread in the population. The daughter cells are born free of super-assemblies and respond to pheromone. Hence, the behavior of these super-assemblies is strikingly opposed to that observed of prions. Indeed, prion proteins are inherited by both the mother and the bud at mitosis and are therefore 'infectious': virtually all cells in the population carry the prion. Due to this difference and to its role in memory, we named Whi3 a mnemon. Another important distinction between mnemons and prions resides in the frequency of their conversion. While in a population of cells not exposed to pheromone nearly no cells have a Whi3 super-assembly, cells treated for three hours with pheromone almost all form a Whi3 super-assembly. Somehow, pheromone induces super-assembly very efficiently. This is not the case for prions, the conversion of which is thought to be stochastic and very rare (conversion arise at a frequency of 10^-6^-10^-7^). Therefore, Whi3-based super-assemblies - mnemons - differ from prions in both their mode of induction and inheritance.

**Figure 1:**
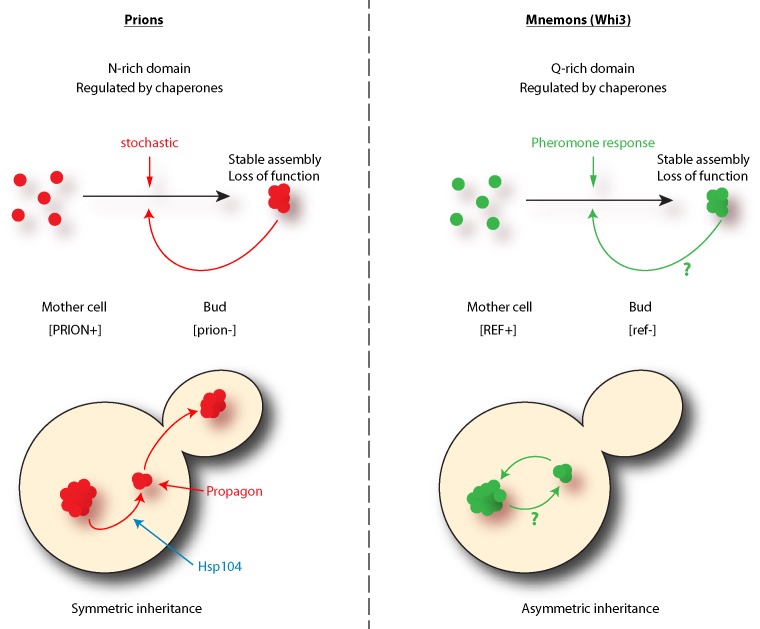
FIGURE 1: Mnemons differ from prions. Comparisons of some of the properties that are shared or unique to prions and mnemons. Note that the main differences lie in the mode of inheritance at mitosis and the induction of super-assemblies. [REF+] and [ref-] denote the pheromone refractory state. Question marks highlight unknown parts of mnemons biology.

## Are most PrD containing proteins mnemons?

*In silico *surveys have highlighted roughly 200 proteins in budding yeast that have a prion-like domain, based on similarities with the known prion domains of Sup35, Rnq1, Ure2 and New1. However, in classical assays for prion conversion, for example overexpression of the prion-like domain fused to a GFP to see the formation of aggregates by fluorescence microscopy or to detect SDS-resistant species biochemically, most of them do not behave as 'canonical prions'. Why is that? Although these proteins contain a stretch of amino acids predicted to drive the protein to form some sort of super-assemblies, there are two considerations to take into account: 1) the formation of these assemblies may not be stochastic, and over-expression might simply not be enough to drive their formation. In addition, these assemblies may contain multiple proteins, allowing their formation only in highly specific situations. 2) if these assemblies form only in a few cells but do not spread mitotically because they are kept in the mother cell, then the probability to identify such cells in an exponentially growing population is very low. Furthermore, prion-like domains are also found in proteins involved in p-body formation as well as nucleoporins. These large complexes are probably driven by the strong interaction platforms provided by the prion-like domains. However, many more proteins contain prion-like domains and many might form super-assemblies that segregate discretely at mitosis. Therefore, we suspect that the list of mnemons will expand soon and rapidly. Interestingly, the high occurrence of prion-like domains in the proteome is not budding yeast-specific but spreads across virtually all eukaryotes. Thus, mnemons and their role in encoding cellular memories is likely to be widespread. We actually already know of two proteins that behave as mnemons outside yeast: the CPEB protein in Aplysia and Orb2, its ortholog in the fruit fly, are proteins containing prion-like domains and involved in long-term plasticity of neurons. Super-assembly of these proteins at synapses is required for memory maintenance. Synapses are compartments, separated from neighboring synapses of the same neurons and it might well be that the formation of these super-assemblies allows each synapse to keep traces of its activation history. Therefore, mnemons may be part of the mechanism of memory maintenance in the brain, suggesting that the same principles might apply from yeast to humans.

